# Swarm Metaverse for Multi-Level Autonomy Using Digital Twins

**DOI:** 10.3390/s23104892

**Published:** 2023-05-19

**Authors:** Hung Nguyen, Aya Hussein, Matthew A. Garratt, Hussein A. Abbass

**Affiliations:** School of Engineering and Information Technology, University of New South Wales, Canberra, ACT 2600, Australia; hung.nguyen@student.adfa.edu.au (H.N.); a.hussein@adfa.edu.au (A.H.); m.garratt@adfa.edu.au (M.A.G.)

**Keywords:** shepherding, digital twin, swarm metaverse, symbiotic simulation, swarm robotics, human-swarm interaction, gestural communication, levels of autonomy

## Abstract

Robot swarms are becoming popular in domains that require spatial coordination. Effective human control over swarm members is pivotal for ensuring swarm behaviours align with the dynamic needs of the system. Several techniques have been proposed for scalable human–swarm interaction. However, these techniques were mostly developed in simple simulation environments without guidance on how to scale them up to the real world. This paper addresses this research gap by proposing a metaverse for scalable control of robot swarms and an adaptive framework for different levels of autonomy. In the metaverse, the physical/real world of a swarm symbiotically blends with a virtual world formed from digital twins representing each swarm member and logical control agents. The proposed metaverse drastically decreases swarm control complexity due to human reliance on only a few virtual agents, with each agent dynamically actuating on a sub-swarm. The utility of the metaverse is demonstrated by a case study where humans controlled a swarm of uncrewed ground vehicles (UGVs) using gestural communication, and via a single virtual uncrewed aerial vehicle (UAV). The results show that humans could successfully control the swarm under two different levels of autonomy, while task performance increases as autonomy increases.

## 1. Introduction

The field of swarm robotics borrows lessons from biological swarms to enable the cooperation of multi-robot systems to carry out complex tasks [1]. A group of individual robots acting cooperatively to achieve a common goal is referred to as a robot swarm. These robots typically have limited sensing and processing capacities. Previous research has demonstrated the capability of swarms of small robots to do a variety of real-world activities, including collective transportation [2], search and rescue [3], surveillance [4], and monitoring bush fires [5]. Robot swarms are needed in missions demanding more performance than what a single robot system can offer. Additionally, resilience, flexibility, and scalability are three key benefits of well-designed swarms over single-robot systems [1]. Resilience is the swarm’s ability to continue functioning even when some of its members fail. Due to their spatial dispersion, swarms are resilient to individual failures, as other swarm members can replace lost or faulty members. Flexibility can be achieved by adjusting the local interactions between swarm members to produce a broad range of swarm-level behaviours. Scalability results from the minimal size-dependent variance in swarm performance [6].

Despite the potential of robot swarms in various application sectors, completely autonomous swarms are not anticipated to be operational in the near future due to numerous technical difficulties and accountability issues [7,8]. This makes the human component essential for swarm operations to ensure that swarm behaviours align with mission goals and to maintain success in challenging circumstances [9].

Human–swarm interaction (HSI) studies the dynamics of interactions between human team members and robot swarms to enhance team performance. Human interaction with robot swarms is challenging due to the limited cognitive capacity of humans relative to both the amount of information generated by swarm members and the number of control signals needed to be sent concurrently to all swarm members [10,11,12,13]. This raises the need for scalable interaction mechanisms where the workload placed on the human does not grow significantly with the size of a swarm.

Since the early days of humankind, humans have managed to control very large swarms of sheep via shepherding, where a human communicates their intent to a sheepdog (control agent), which in turn chases swarm members to guide them along a path dictated by the human. Using a sheepdog facilitates controlling the swarm; thanks to the cognitive and physical superiority of the dog compared to the swarm members. Inspired by sheep herding, the concept of shepherding has been reproduced in robotic systems and has demonstrated its potential as a scalable mechanism for swarm control [14].

Nevertheless, existing mechanisms for HSI, including shepherding, have mostly been developed and evaluated in simulated environments with minimal guidance on how to transfer these mechanisms to the control of physical robot swarms. The complexity of implementing HSI mechanisms in the real world slows down the advancement of this research area. For example, shepherding implementations require the availability of a physically and cognitively superior control agent, which imposes extra costs for the acquisition, maintenance, and personnel training required to operate the control agent. Any failure or loss of this control agent renders the whole swarm mission unattainable, creating a very costly environment to run, especially in an academic context running on a tight budget.

Interestingly, the concept of a *metaverse*, where the physical and virtual worlds are combined, may hold a proper solution to this issue. Our proposed metaverse incorporates a real-world component where the physical swarm interacts with its environment and a symbiotic simulation component containing digital twins for the swarm members. The parameters of the digital-twin swarm are updated and uploaded through a real-time data link with the real world. One or more virtual control agents are employed in the simulation component, through which the human can guide the swarm using shepherding guidance tactics. The user sees the world through a graphical user interface (GUI), such that the human could be unaware of what is physical/real and what is simulated/virtual. The main advantage of the proposed metaverse are twofold. First, it is a cost-effective solution to test shepherding as a swarm guidance tactic while waiving some of the expensive platforms that represent the physical control agent. Second, it makes it feasible to use powerful AI concepts as part of the virtual control agent to support high levels of autonomy.

To the best of our knowledge, this is the first study to propose a digital-twin-enabled metaverse for scalable HSI. The contributions of the paper can be summarised as follows:

- Proposing a digital-twin enabled metaverse for scalable control of physical robot swarms by utilising the shepherding control strategy with a virtual control agent.
- Implementing the proposed metaverse in the control of a physical swarm of Turtlebot-3 UGVs. This is enabled by coupling the physical environment with a simulated Gazebo environment where the digital-twin swarm and the virtual control agent operate.
- Implementing two levels of autonomy (LoA) for the interaction between the human and the virtual control agent, and designing two gesture communication languages to support these LoA.
- Validating the proposed metaverse by presenting the results of using it to control the physical swarm under two LoA.

The rest of the paper is organised as follows. Section 2 reviews three areas: HCI control mechanisms, levels of autonomy in HSI, and gestural communication. The design of the metaverse is then presented in Section 3. This is followed by a case study in Section 4 to test the proposed framework and its use in controlling physical swarms. Conclusions are then drawn in Section 5 with some discussion points and concluding remarks. Suggested directions for future work are finally presented in Section 6.

## 2. Related Work

Robot swarms, as with other types of automation, lack the human-like intelligence required to handle unforeseen circumstances [15]. Because the majority of swarm studies to date have been conducted in controlled lab settings, the performance characteristics of swarms have also not been thoroughly tested in realistic circumstances. As a result, it is anticipated that human engagement will be necessary for swarm operations for the foreseeable future [9]. Numerous studies have demonstrated that the presence of humans can be advantageous or even necessary for the performance of swarm activities [7,9]. Robots can swiftly and accurately complete repetitive and specialised jobs, whereas humans can deal with dynamic and unstructured environments because of their superior cognitive capacities. Combining human and robotic abilities can increase the likelihood of successful completion of complex missions. This section discusses related work on a number of key design aspects affecting HSI performance. We present existing mechanisms for HSI and discuss their limitations. We then describe the roles that should be assigned to humans in HSI by discussing the impact of different levels of autonomy on team performance. Finally, we discuss control modalities with a focus on gestural communication as an intuitive channel for sending human commands to the swarm.

### 2.1. HSI Control Mechanisms

The creation of mechanisms that permit smooth interactions between humans and swarm members is a major issue that limits the potential of human–swarm teams [8,9]. The most basic approach to swarm control is by tele-operating individual swarm members. Earlier studies investigated the effect of the human-to-robot ratio on team performance in multi-robot systems [16,17]. For example, in simulated search and rescue missions, it was found that increasing the number of robots in the system from four to eight improved task performance, but then increasing the number of robots to twelve caused a performance drop [18]. This was explained by the monotonic increase in human workload and decrease in robot-level performance as more robots were controlled by a human [18]. The scalability challenge is exacerbated in swarm systems, as individual robots are no longer independent; instead, their inter-dependencies lead to an exponential increase in control complexity as a function of the number of robots [7].

Studies on HSI sought to address the scalability issue by proposing interaction mechanisms that take the inter-dependencies between swarm members into account. Table 1 provides a summary of the existing swarm control mechanisms. Kolling et al. [7] identified four classes of swarm control mechanisms: (1) *behaviour selection*, (2) *parameter selection*, (3) control via *environmental signals*, and (4) control through *few swarm members*. Swarm control via *behaviour selection* requires the existence of a library of behaviours for the human to select from such that the human acts as a supervisor who can intermittently interact with the swarm to switch between its behaviours. Kolling et al. [19] investigated the use of *behaviour selection* where a human can select a subset of the swarm and issue a command for behaviour switching to one of seven programmed behaviours. The results showed that novice human operators could easily use *behaviour selection* for swarm control. Walker et al. [20] used *behaviour selection* to offer a high level of autonomy for the swarm, which they showed to be important in obstacle-cluttered environments. Nagavalli et al. [21] studied the use of *behaviour selection* to achieve a given swarm formation (V, spiral, or circle) in shape-changing tasks. The timing of issuing the behaviour switching command was shown to be critical for task success; however, the human operators were able to learn to improve the timing of their commands over time [21]. More recent studies [22,23] used *behaviour selection* in controlling swarms of UGVs; however, behaviour switches are triggered autonomously with no human input.

Overall, as a mechanism for swarm control, *behaviour selection* has a number of advantages, including high levels of autonomy and ease of use by novice human operators. Nonetheless, *behaviour selection* requires all swarm behaviours to be programmed ahead, and it lacks adaptability to changing environmental conditions [19]. Moreover, *behaviour selection* is sensitive to the human operator’s perception of the swarm state and the timing of issuing a command for behaviour switching [21].

*Parameter selection* is another class of control method that aims to give the human more control over the behaviours executed by a swarm. Similar to *behaviour selection*, the set of allowed models for swarm interaction needs to be programmed offline. However, the swarm models can be parameterised in real-time by the human operator to allow for more flexibility. Behaviour parameterisation can enable a wide range of swarm operations; for instance, Couzin et al. [24] showed that their swarm model could be parameterised to generate variants of the aggregation, torus, and flocking behaviours. However, control by *parameter selection* can be very difficult, as it indirectly affects the swarm’s emerging properties. Usually, parameter selection is performed at the stage of system development to choose a suitable parameter setting for a given task, either by manual parameter selection [6] or through optimisation [25].

Swarm control via *environmental signals* enables a human supervisor to insert environmental influences for spatial control. That is, the control is applied to swarm members within the region of the environment where the influence is inserted. Previous studies have investigated variants of control by *environmental signals*. Kolling et al. [19] used a combination of *environmental signals* and *behaviour selection* for swarm control. In their work, the human supervisor would select one of the available behaviours and send it to one of the beacons in the environment. Beacons are dynamically placed by the supervisor, who also configures their range. A beacon would keep transmitting the human command it received so that swarm members passing through its range can listen to the command. Kolling et al. found that the performance of control via beacons was inferior to the case where the supervisor directly selects a sub-swarm to execute the command.

Pheromones have also been used as a bio-inspired mechanism for swarm control via *environmental signals* [26,27]. Pheromones can be inserted in the environment by swarm members as a form of information exchange or by a human operator. When inserted, pheromones vanish gradually over time and travel to nearby locations. These characteristics can make it difficult for humans to use pheromones for controlling a swarm. Previous studies have only used pheromones in fully autonomous swarms with no human interventions. Additionally, it has been shown that pheromones do not scale well with swarm size [27]. Kim et al. [28] studied chemotaxis control, where a chemical source is placed in the environment to guide the swarm members. The chemical concentration decreases over distance, making it detectable only within a certain region. Chemotaxis control was shown to be successful at guiding robots towards the designated locations. However, all the experiments were conducted in simulation and in a fully autonomous manner with no human input during the task.

The last class of swarm control mechanisms is control via *few swarm members*, where the human sends their commands (low-level tele-operation [29] commands or high-level guidance) to one or more swarm members. Agent-to-agent interaction allows the propagation of human commands from the few controlled agents to the rest of the swarm. The controlled agents can be leaders that other agents follow [29], predators that other agents repel from [30], or just normal agents that other agents interact with indistinguishably [31]. The vast majority of studies have used control by leaders rather than control by predators or normal agents. Control via a single agent has received the most interest in the literature [29,32], although a few studies have considered swarm control by a human via multiple agents [37].

Overall, control via *few swarm members* has been validated by several studies as a useful mechanism for controlling simulated swarms [30,37]. However, its validation with physical swarms has been very limited, e.g., [38]. Control via *few swarm members* is the most promising mechanism for HSI. It allows the human greater flexibility than *behaviour selection* in intervening with swarm operations in response to unforeseen conditions, it avoids the difficulty of selecting the right combination of parameters needed by *parameter selection*, and it gives the human more direct control over individuals and sub-groups of the swarm than *environmental signals*.

One of the key concerns of using control via *few swarm members* is that identifying these control members enables an adversary to take control over the whole swarm [39] by compromising or deceiving these members [33,34,36]. For example, leader identification can be performed by a human or using machine learning techniques [35]. A few studies have proposed algorithms for hiding the leader’s identity [35,40]; however, the risk of correct identification is still very high [35]. This means that using just a few of the swarm members to control the others increases the vulnerability of the swarm. Our proposed metaverse aims to address this challenge by using a symbiotic simulation environment with *virtual* control agent(s) such that the behaviour of the virtual control agents eventually influences the physical swarm members.

Another gap in the literature relates to the type of control agents. The vast majority of HSI studies used leaders for control, which typically assumes that swarm members are clustered in one group such that the leader’s influence can easily propagate between swarm members. Nonetheless, many tasks require the swarm to be scattered during some phases, such as in environmental monitoring [41] and target search [42]. Therefore, the type of controlled agents needs to allow for controlling the swarm under different clustering conditions. In our proposed metaverse, we utilise a sheepdog model for the control agent and a sheep model for swarm members. This bio-inspired control method is selected not only for its scalability as demonstrated in natural and simulated swarms [14], but also for its ability to control swarm members under various clustering conditions [43].

### 2.2. Levels of Autonomy

Different roles can be assigned to humans in HSI. Scholtz [44] presents five roles that people can play when they interact with robots: supervisor, operator, teammate, bystander, and mechanic. The supervisor’s primary duty is to assess the overall situation in light of mission objectives and provide mission-level guidance or alter high-level plans. On the other hand, low-level action jobs, such as motor control, require the direct control of a human operator. As a teammate, a human collaborates with robots to accomplish mission goals and gives the robots high-level directives without changing the overarching mission objective. The positions of the bystander and mechanic are the last, and they have less to do with our area of concern. A bystander does not directly interact with the robot; however, their presence may affect a robot’s low-level behaviours, e.g., collision avoidance. A mechanic physically interacts with robots to maintain their physical components.

Human performance is improved when they function as supervisors rather than operators, according to previous HSI studies [8,45]. On the one hand, when acting as an operator to control low-level swarm actions, a human can exhibit inferior performance due to exhaustion, task-switching-related distractions, and decreased situational awareness [46]. On the other hand, performing the role of a supervisor or a teammate enables a human to effectively interact with the swarm by combining the strengths of humans and robots. For instance, Kolling et al. [47] discovered that even simple robot swarms operating in a fully autonomous manner outperformed humans in simulated foraging operations. However, they found that humans were better at adapting to complex situations. Thus, it is advantageous to assign humans more supervisory responsibilities in order to improve mission performance.

### 2.3. Gestural Communication

Designing easy-to-use interfaces is an important factor affecting the performance of human–robot teams [48]. Classical robot interfaces mainly used keyboards, push buttons, or joysticks for input commands. Such interfaces limit human mobility during the interaction and require specialised training, which may be time consuming and undesirable [49]. With the increasing adoption of robots in a wide range of domains (e.g., service and healthcare robots), there has been a growing focus on intuitive interfaces that are easy to use by lay people with minimal training requirements [50]. Thus, the interest in designing natural interfaces, e.g., speech [51] and gesture interfaces [52], has grown recently. Communication among people often involves gestures. For instance, pointing to an object is much easier than using spoken language to describe its exact location. Therefore, gesture-based interfaces have drawn significant attention in the area of human–robot interaction.

Gesture interfaces have been successfully applied in HSI settings. Podevijn et al. [53] designed an interface for swarm control in resource allocation tasks using gestures. The interface included a set of gesture vocabularies corresponding to high-level commands: select, steer, stop, merge, and split. When a human selects one of these high-level commands, the command is mapped to the corresponding swarm behaviour from a library of programmed behaviours. The interface was shown to enable humans to select subswarms and direct them towards different locations. Alonso-Mora et al. [54] proposed a gesture-based interface that allows human control of robot swarms in interactive display scenarios. The interface enabled different modes of swarm control, allowing the user to issue commands for individual robots after selecting them or issue commands to the entire swarm, for example, by using gestures to select one of the pre-defined shapes that the swarm can form.

The gesture interface proposed in [54] allows the human to select individual robots or subswarms and set their trajectories. Different combinations of right-hand and left-hand gestures are used for different purposes, such as robot selection and trajectory or formation setting. Chen et al. [55] used a multiple-channel interaction by combining speech recognition with gesture communication. In their work, speech is used to provide instructions that operate as high-level activities, whereas gestures are used for selecting virtual drones to execute the instructions.

To ensure the effectiveness of gesture interfaces, it is important to design intuitive gestures that do not impose a significant physical workload on the human. Rempel et al. [56] provide guidance on how to design comfortable hand gestures that do not cause significant hand pain and fatigue. Their research is based on lessons learnt from designing gestures for sign languages. They concluded the following five guidelines for designing comfortable hand gestures:

- Regularly performed tasks should be given to gestures that are easy to repeat, whereas less-frequent tasks can be given less-comfortable gestures.
- Comfortable gestures are those where the hands are close to the lower chest region and the body’s mid-line without being too far from the torso. The hands should not be wider or higher than the shoulders and no more than 45 degrees of shoulder flexion should be allowed to separate the hands from the body.
- Gesturing should not be required at a high rate and should not include hands striking against each other or against hard objects.
- Postures and motions for the wrist and forearm should be neutral. The set of comfortable wrist and forearm gestures is listed in [56].
- Postures and motions for the hand and fingers should be relaxed and should avoid the need for full extension.

## 3. Digital-Twin Metaverse for Swarm Control

**Figure 1:**
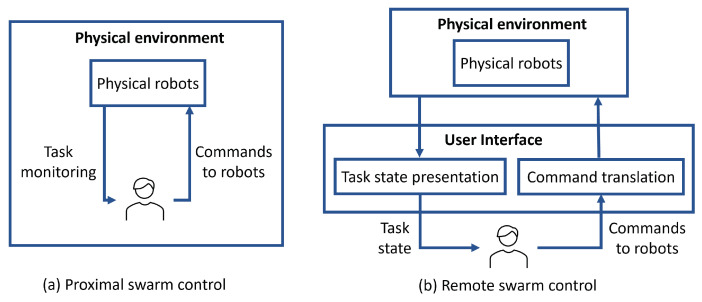
Proximal (**a**) and remote (**b**) swarm control techniques.

Swarm control can either be proximal where the human co-exists with the swarm in the physical environment [57], or remote where the human is deployed in a different environment and interacts remotely with the swarm by monitoring their state and sending commands over a communication network [58]. Proximal control, shown in Figure 1a, is not always feasible, particularly in risky or inaccessible environments.

Traditional remote control settings, shown in Figure 1b, are preferred due to their suitability for a wide range of operations as they only require the existence of a communication infrastructure that links between the human and the swarm environments. In remote control settings, the human is provided with a user interface that enables them to monitor the swarm state, e.g., using a GUI, and send commands. Command translation could be required to map human input controls (e.g., voice commands, gestures, or joystick inputs) to robot-interpretable commands. In both proximal and remote swarm control, human commands can be sent to all the swarm members (e.g., using behaviour switching control), to individual robots (e.g., leaders), or disseminated via environmental signals (e.g., using pre-located beacons). Each of these control methods has its advantages and limitations as discussed in Section 2.

**Figure 2:**
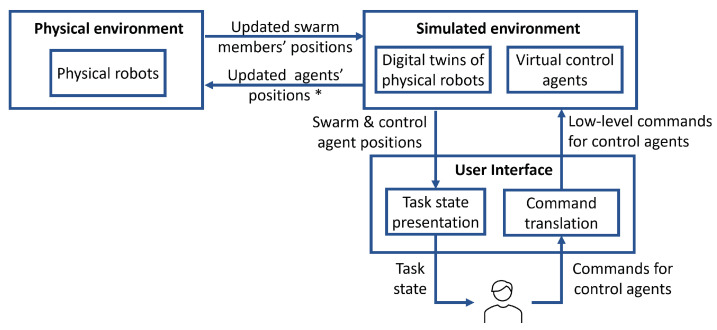
The proposed digital-twin-enabled metaverse for swarm control. * Updated agents’ positions can be the updated positions of the control agents or of the swarm members, as discussed in Section 3.

Figure 2 provides an overview of the proposed metaverse that aims to address the limitations of the control methods afforded by traditional proximal and remote control settings. The metaverse consists of three main components: the physical environment, the symbiotic simulated environment (or the simulated environment for short), and the user interface. The physical environment is where the robot swarm is deployed to achieve a given mission goal. Swarm robots can sense their physical environment using their onboard sensors and can act upon it using their actuators. The second component is the simulated environment, which contains a digital twin for each member of the swarm, along with one or more virtual control agents. A digital twin is ideally an accurate model of the physical robot it represents, whereas a virtual control agent can be any robotic platform that allows the human to flexibly exert control over the swarm members. The third component is the user interface—it contains a module for task state presentation (e.g., visualisation) to allow the human to monitor the task and a module for command translation.

The control flow is shown in Algorithm 1 and can be described as follows. The physical robot swarm is deployed in the operation environment to achieve a given goal. The simulated environment is initialised with an equal-sized swarm of digital twins in addition to one or more virtual control agents (lines 1–3). The models of the digital twins are set based on $\Pi_{models}$ to provide replicas of the physical robots. The desirable models of the virtual control agents are specified as input variable $\beta_{models}$. For each time step, the positions and headings of the physical robots are estimated and sent to the simulated environment such that the digital twins’ positions are adjusted accordingly (lines 6–7). The initial positions of the virtual control agents can be set by the human operator. The human monitors the task state and sends their commands to the control agents (lines 8–9). The commands should be designed to allow for a desirable level of autonomy, for example, the human can tele-operate the control agents or send them higher-level commands. Human commands are translated into robot-interpretable commands and sent to the simulated environment (line 10). The commands are executed in the simulated environment such that the positions of the control agents are updated (line 12).

Each digital-twin robot senses the new positions of the control agents and determines its movement according to its programmed decision rules (line 14). Based on the design decision, the simulated environment can send the physical environment either the new positions of the digital twins or only the new positions of the control agents, leaving it to the physical robots to determine their next movement (lines 15–23). This design decision would depend on the available communication resources. In either case, and after action execution, the position and orientation of each physical robot are estimated and sent to the simulated environment such that the position and orientation of the corresponding digital twin are set accordingly. The next subsection provides more details on the role of digital twins in the proposed metaverse. This is followed by a discussion on setting the level of autonomy in the interaction between the human and the control agents, and a description of the shepherding model to facilitate the interaction between the control agents and members of the swarm.
**Algorithm 1** Swarm control in the proposed metaverse**Input:** 
$\Pi_{models}$, $\beta_{models}$, $T_{max}$, send_digital_twin_data
   1:$\Pi_{twins}$← initialiseDigitalTwins($\Pi_{models}$)   2:β← initialiseVirtualControlAgents( $\beta_{models}$)   3:t ← 0   4:**while** t $\leq T_{max}$ **do**   5:    t ← t + 1   6:    $x_{phys},y_{phys},\theta_{phys}$← getPhysicalSwarmData( )   7:    updateDigitialTwinsData($\Pi_{twins}$, $x_{phys},y_{phys},\theta_{phys}$)   8:    updatedGUI($\Pi_{twins}$, β)   9:    ς← getHumanCommand( ) 10:    ζ← translateCommands(ς) 11:    **for** i in length(ζ) **do** 12:        executeCommand(β[i],σ[i]) 13:    **end for** 14:    applySheepBehaviours($\Pi_{twins}$,β) 15:    **if** send_digital_twin_data **then** 16:        sendDigitalTwinPositionsAndHeadings($\Pi_{twins}$) 17:        updatePhysicalRobotPositions( ) 18:    **else** 19:        sendControlAgentPositions(β) 20:        **for** each physical robot **do** 21:           applySheepBehaviour( ) 22:        **end for** 23:    **end if** 24:**end while**


### 3.1. Role of Digital Twins

The role of the digital twins in the simulation environment can be explained as follows. The interaction between the control agent and the digital twins acts as a safety-net (i.e., real-time verification module) that allows for verifying the impact of human commands before executing them in the physical environment. For example, the detection of potential safety risks (e.g., collisions) among the digital-twin robots could be used to prevent the execution of commands in the physical environment. Thus, it is important to frequently update the digital twins’ position and orientation information using their physical counterparts to continuously calibrate the digital twins when deviations occur in the physical environment. This will help avoid the accumulation of errors due to approximations and will ensure that interactions happening in the simulated environment closely align to those occurring in the physical environment.

### 3.2. Levels of Autonomy in Human-Control-Agents Interaction

In our proposed metaverse, the human indirectly influences swarm operation via the control agents. The human interaction with the control agents can have different levels of autonomy. On the one hand, for the lowest level of autonomy, a human would tele-operate the control agents and direct their low-level actions. Low levels of autonomy could impose a significant workload on the human as, even with one or few control agents, the human still needs to observe and assess the states of all the swarm members, which can be very cognitively demanding. On the other hand, the highest levels of autonomy would allow the human to communicate the task objective to the control agents and then have no further interactions with them till the end of the task. Such fully autonomous operation is, however, not possible in realistic environments given the current state of technology [59] and is not desirable for ethical and accountability issues [15].

Mid-level autonomy would allow the human to have more influence whilst not fully controlling the control agents. This can be very useful to ensure the human stays within the control loop while avoiding increasing their cognitive load. Semi-autonomous swarm operations with mid levels of autonomy can be operationalised in different ways within the proposed metaverse. For example, a human can select high-level behaviours for each control agent using a library of pre-defined behaviours, can assign sub-swarms to different control agents, or can command the control agents using waypoints. Designing a suitable set of commands for controlling the control agents is of the utmost importance to ensure the human can effectively control the swarm. As discussed in Section 2.1, the commands should enable the human to control the swarm members with different grouping configurations. This would enable reusing the commands across various task domains. We propose using the shepherding model to control large numbers of swarm members under different grouping configurations using a few control agents. Below is a detailed description of the shepherding model and its use in our metaverse.

### 3.3. Shepherding Model

Shepherding is a bio-inspired strategy for the guidance of large swarms using a few control agents. In shepherding, a human uses a dog β to influence the movement of a flock of *n* sheep $\Pi =\{\pi_{1},\pi_{2},\ldots ,\pi_{n}\}$ and guide them towards a given destination. The operation of an autonomous dog, β, can be described in terms of two behaviours: collecting and driving. The collecting phase is activated when swarm members are not clustered in one group. To collect the sheep, β finds the swarm member $\pi_{i}$ furthest from the geometric centre of mass *GCM* of the flock and collects $\pi_{i}$ towards the *GCM*. This is achieved by setting the dog’s target position as follows:

(1) $$P_{\beta }^{t+1}=P_{\pi_{i}}^{t}+R_{\pi \beta }.\frac{P_{\pi_{i}}^{t}-GCM^{t}}{∥P_{\pi_{i}}^{t}-GCM^{t}∥}$$

such that $P_{x}^{t}$ is the position of agent *x* at time *t*, and $R_{\pi \beta }$ is the distance within which a swarm member can sense the presence of β. The dog keeps collecting astray sheep towards the centre of mass till all swarm members form one cluster. This swarm state triggers the driving behaviour, where the dog moves behind the GCM of the sheep flock such that the flock becomes the middle between the dog and the target destination, denoted by $P_{dest}$. This is achieved by setting the dog’s target position using the following equation:

(2) $$P_{\beta }^{t+1}=GCM^{t}+R_{\pi \beta }.\frac{GCM^{t}-P_{dest}}{∥GCM^{t}-P_{dest}∥}$$

A sheep agent $\pi_{i}$ is repelled from the dog β when β is within a distance of $R_{\pi \beta }$ from $\pi_{i}$. Additionally, $\pi_{i}$ moves towards the geometric centre of mass of its sheep neighbours that are within its sensing range $R_{sense}$ and is repelled from the geometric centre of mass of very close neighbours, i.e., those within $R_{\pi \pi }$, to avoid colliding with them. These sheep behaviours are then linearly combined to calculate the next target position for $\pi_{i}$.

In the proposed metaverse, the virtual control agents assume the role of the dogs that can be controlled by the human to guide the swarm members (sheep). As discussed in Section 3.2, the interactions between the human and the control agents are designed based on the selected level of autonomy. Meanwhile, the interactions between the dog and the swarm members are defined based on the sheep model, which specifies how a swarm member should respond to the state of the control agents and the other neighbouring swarm members.

## 4. Case Study

**Figure 3:**
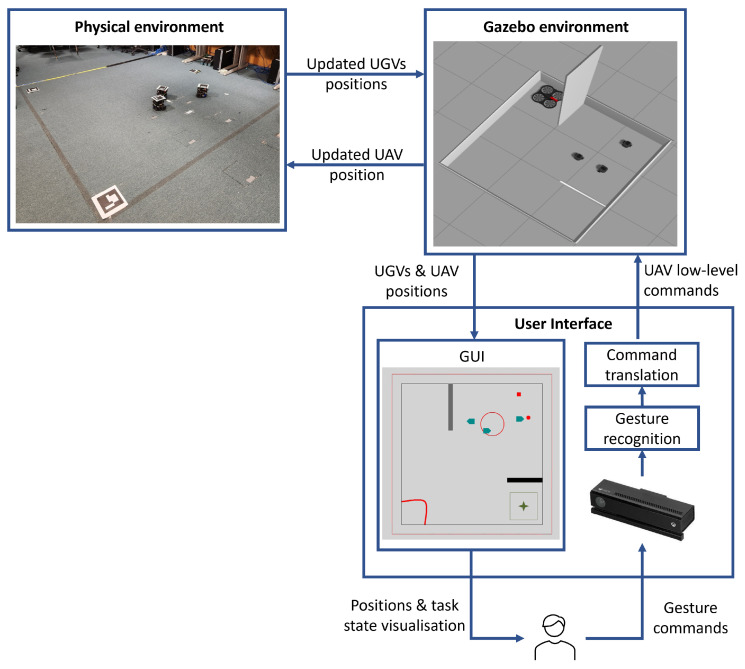
Implementation of the digital-twin-enabled metaverse as used in the case study.

**Figure 4:**
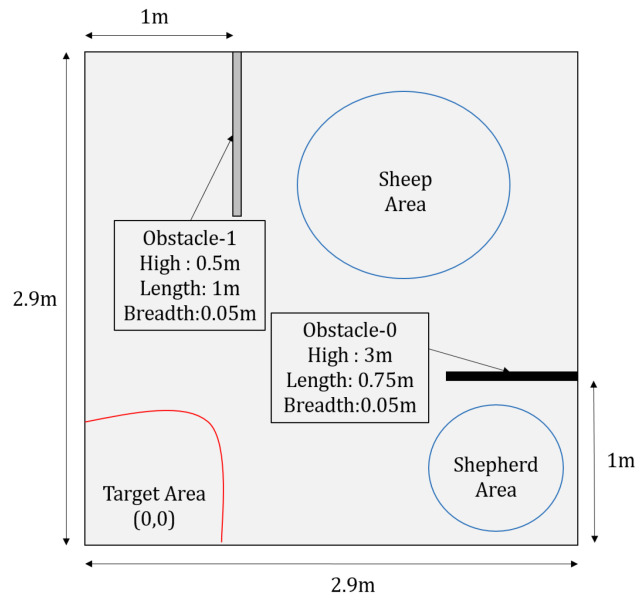
Schematic diagram of the task.

To validate the proposed metaverse, we present a case study where we implemented and tested the metaverse in a swarm control task, as shown in Figure 3. The task starts with three UGVs deployed in the physical environment with the aim of guiding all of them towards a predefined target area. The physical environment is free from obstacles; however, there are constraints on the paths they can traverse reflecting no trespassing areas. Figure 4 gives a schematic diagram of the task where obstacles represent the no trespassing regions. The details of each component of Figure 3 are presented in the following subsections.

### 4.1. Physical Environment

**Figure 5:**
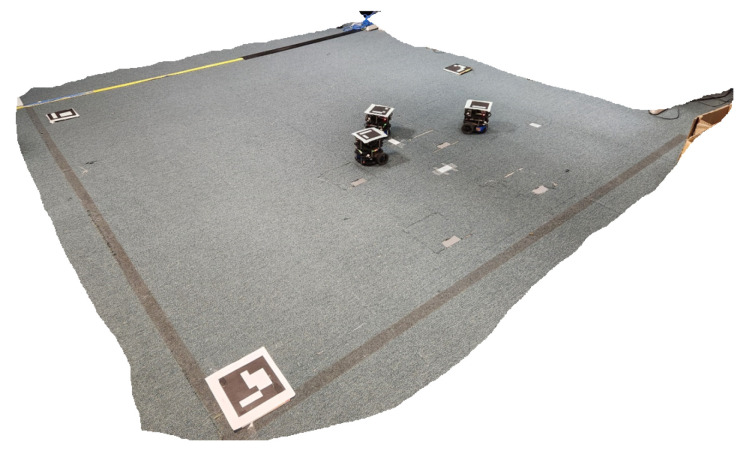
Physical environment.

The physical environment is a square environment with a side length of 2.9 m. Three physical Turtlebot-3 UGVs have been deployed in the environment. Each UGV is equipped with a Raspbian Jessie single board computer (SBC) with a Rasberry Pi 2 processor. The SBCs run Raspbian Linux operating system with ROS Kinetic in order to support communication with other devices. An overhead camera has been used to calculate the positions and orientations of the UGVs, similar to the positioning system used in [60]. To achieve accurate position and heading calculation, Aruco markers have been placed on top of the UGVs as well as at two reference points in the physical environment. Two CAT S62 Pro mobile phones running the Aruco positioning software available in [61] have been mounted on top of the environment to give full coverage of it. Figure 5 shows the physical environment with the three UGVs. ROS Core has been utilised to enable communication between the different devices and components in the system.

### 4.2. Symbiotic Simulation Environment

**Figure 6:**
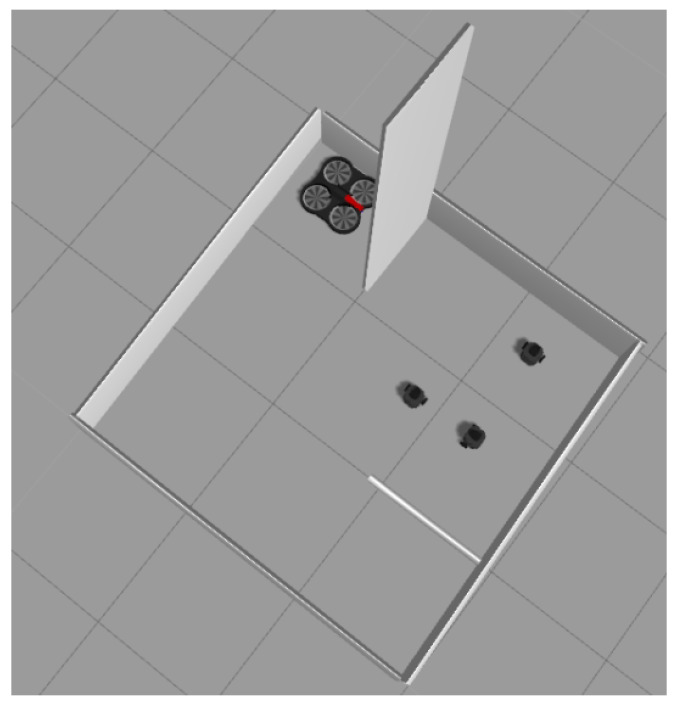
The simulated environment in Gazebo 9.

The symbiotic simulation environment has been implemented using Gazebo 9, a high-fidelity simulator containing models for various commercially available robotic systems. Gazebo 9 was running on a desktop computer with Ubuntu 18.04 LTS and ROS Melodic-Desktop-Full. The simulated UAV model employed in this work is the open-source AR.Drone 2.0 from the TUM simulator [62]. A rotary-wing drone is controlled by four low-level control actions, including thrust, roll, pitch, and yaw. The UAV has been used as the virtual control agent. The UAV controller is a Python script that receives low-level commands using TCP/IP communication and then passes them to the UAV. Turtlebot-3 simulation models have been used as the digital twins for the three physical UGVs. A screenshot of the simulation environment is shown in Figure 6. It can be seen that the simulation environment contains walls that do not exist in the physical environment. These walls are placed to prevent the UGVs from going into certain areas as defined by the task owner. That is, the design of the structure of the simulated environment can facilitate enforcing some constraints on the UGVs’ paths.

### 4.3. GUI

**Figure 7:**
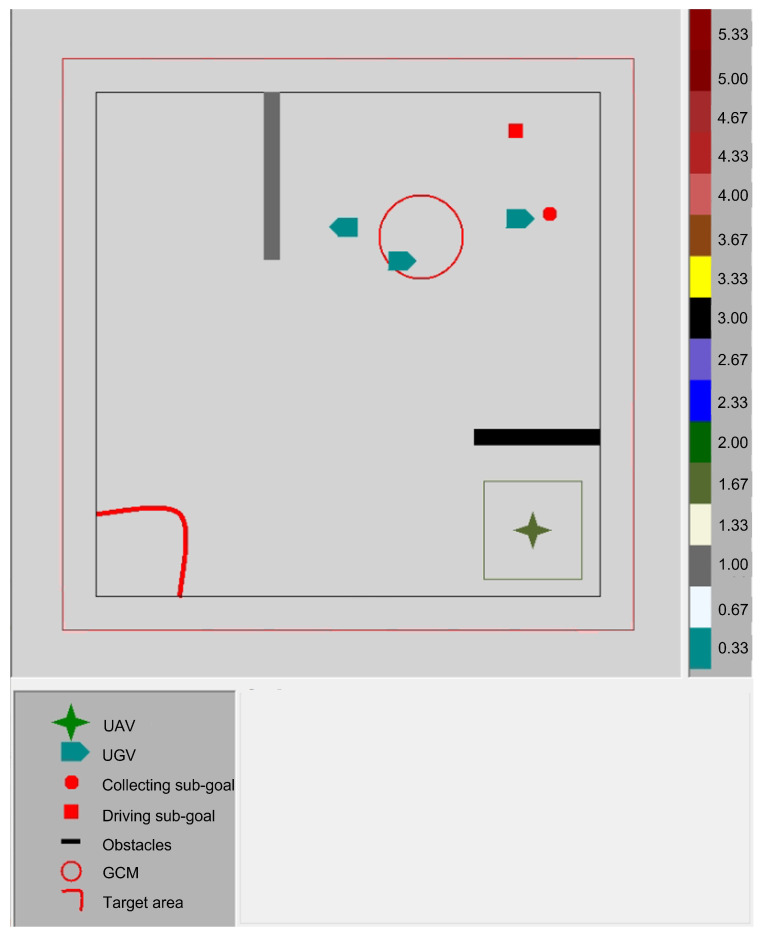
The GUI used in the case study. The right-hand side shows the colour coding for the height of the UAV.

The GUI provides visualisation of the task state including the position and heading information of the UAV and the UGVs as well as supporting information to assist the human with controlling the swarm. The GUI is written as a C# program and is designed to be in real-time so that the operator is able to track the movement of the entire swarm precisely before producing control gestures for the UAV. Figure 7 shows the GUI used in our implementation. The x,y positions of the UGVs and the UAV are plotted in the 2D visualisation environment, whereas the height of the UAV is visualised by changing its colour based on the colour coding provided at the right side of the GUI. Depending on the level of autonomy used and the set of commands available to the human, the GUI can visualise supportive information to give the human insights into the swarm state and task progress. In our implementation, information related to the centre of mass of the UGVs, the collection sub-goal, and the driving sub-goal are presented to the human. The GUI can also be used to visualise task-related information, for example, the target area that that UGVs need to go to.

### 4.4. Levels of Autonomy and Gestural Commands

We implemented two levels of autonomy to allow for different levels of human influence on the UAV operation. In the low level of autonomy case, the human controls the UAV by continuously sending commands to control the altitude (up, down, and maintain the same altitude) and the movement in the x-y plane (forward, backward, left, right, and hover). In the high level of autonomy case, the human sends one of these high-level commands: collect, drive, and hover. The collect command directs the UAV to autonomously apply the algorithm for collecting astray UGVs towards the GCM of all the UGVs (denoted by collection sub-goal in Figure 7). Similarly, the drive command directs the UAV to go behind the UGVs’ GCM and drives it towards the driving sub-goal. The human needs to tell the UAV whether the driving and collection sub-goals should be re-computed to avoid having them move outside of the environment or to avoid potential collision among the UGVs. In addition, the human should command the UAV to activate obstacle avoidance when needed.

**Figure 8:**
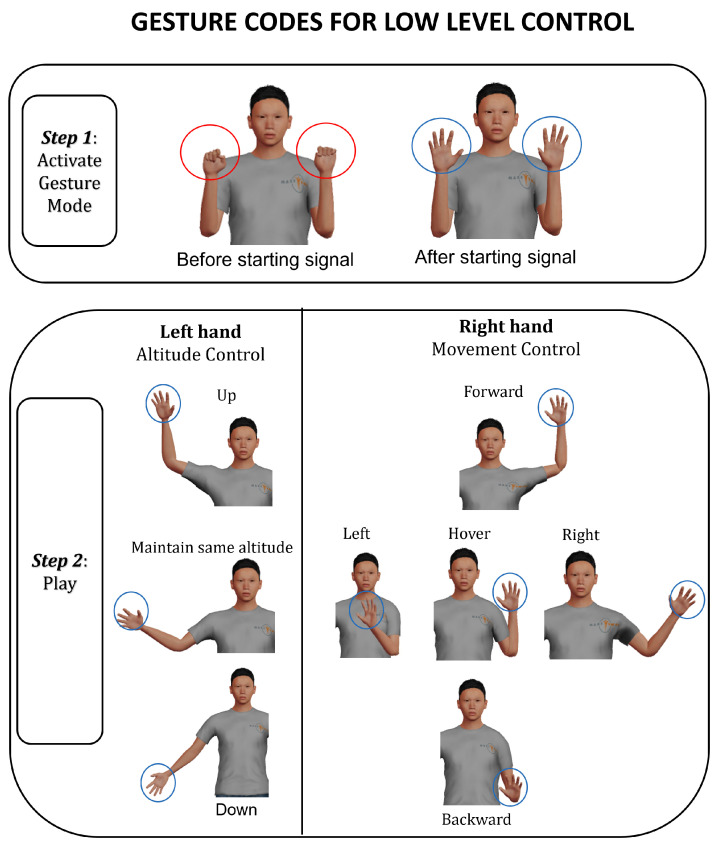
Low level gestures.

**Figure 9:**
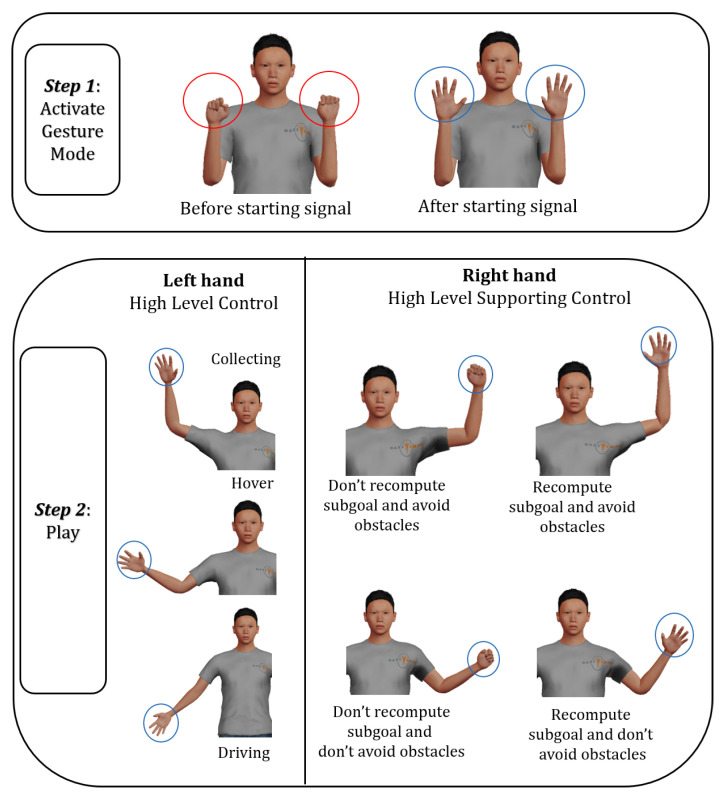
High level gestures.

Gesture communication has been utilised to enable intuitive human interaction with the UAV. We designed two sets of gestures, each serving one of the two levels of autonomy described above. Figure 8 and Figure 9 show the gestures used for the low and high levels of autonomy, respectively. For the low level of autonomy case, the left-hand gestures are used to give commands for altitude changes, whereas the right-hand gestures are used to give commands for movement in the x-y plane. For the high level of autonomy case, the left-hand gestures are used to select between collect, drive, and hover commands, whereas the right-hand gestures are used for signalling the need to recompute collect/drive sub-goals and the need to activate the obstacle avoidance routine. Microsoft Kinect has been used for capturing the gestures and a C# program was developed to recognize the gestures and send them to Gazebo using TCP/IP communication.

**Figure 10:**
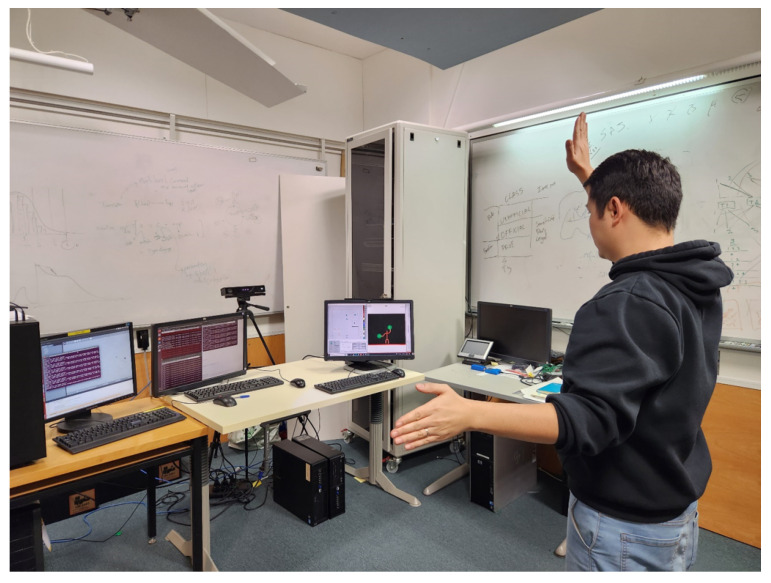
Human control.

Both sets of gestural commands have been designed based on the guidelines presented in [56]. Their gestures are close to the chest region as well as the mid-line of the human body, and around 45 degrees of the shoulder flexion. The postures of the wrist and forearm of both hands are comfortable as they do not require wrist twists and the forearm moves only in the vertical direction. The finger gestures are simple with open-hand gestures not requiring full extension. Figure 10 shows an example of controlling the UAV using gesture commands.

### 4.5. Experiments

We conducted experiments where human operators (first and second authors) performed the task under the two levels of autonomy. For each level of autonomy, each operator underwent a training session to become familiar with the gestures. Each training session consisted of ten episodes where the swarm was fully simulated (i.e., there were no physical UGVs) such that the task is to guide all the simulated UGVs to the target area. The decision to run the training sessions in a fully simulated environment is to focus the human attention on learning the gestures without becoming occupied or confused by the complexity of controlling physical UGVs and the associated actuation errors that occur in a real environment. After each training session, the human performed twenty episodes under the same level of autonomy where the task involved both the simulation and physical environments. The mission objective was to use the virtual UAV to guide all the physical UGVs inside the target area. It is worth noting that the interface, including the GUI and the gestural communication, is agnostic to whether the task is completed in the simulation environment, the physical world, or in a mix of simulation and physical settings.

### 4.6. Evaluation Metrics

Four metrics have been used for evaluating the performance of the proposed metaverse as listed below:

- **Success rate (%)** is the percentage of successful mission completions, such that the mission is considered successful if the GCM of the UGVs is inside the target area within a maximum of 10,000 simulation steps.
- **Number of steps** is the number of steps taken before the GCM of the UGVs becomes inside the target area. This metric reflects the time efficiency of the mission.
- **UGVs travel distance (m)** is the average total distance travelled by the three UGVs. This metric reflects the energy efficiency of the mission.
- **Number of switches** is the average number of switches between gestures. This metric reflects the psycho-physical load imposed on the human due to the required rate of issuing commands. Fixing commands is easy to manage mentally, but will put a restraint on the joints repeating the commands to the robot, while fast switching commands could increase both cognitive and physical loads.

### 4.7. Results

Table 2 shows the success rate (SR) as well as the mean μ and standard deviation σ of each of the number of switches, number of steps, and UGVs travel distance as calculated from successful episodes. It can be seen that the achieved SR under high level of autonomy (100%) is considerably higher than SR under the low level of autonomy (75%). As for the number of steps, the low level of autonomy recorded notably higher values (mean = 6638, std = 1132) than the high level of autonomy (mean = 5121, std = 1381), with the difference being statistically significant (*p* = 0.015). That is, considering only successful episodes, the low level of autonomy required significantly more time than the high level of autonomy. Similarly, the low level of autonomy had a considerably higher number of gesture switches (mean = 221, std = 66) than the high level of autonomy (mean = 40, std = 13), with the difference being statistically significant (*p* < 0.001).

**Figure 11:**
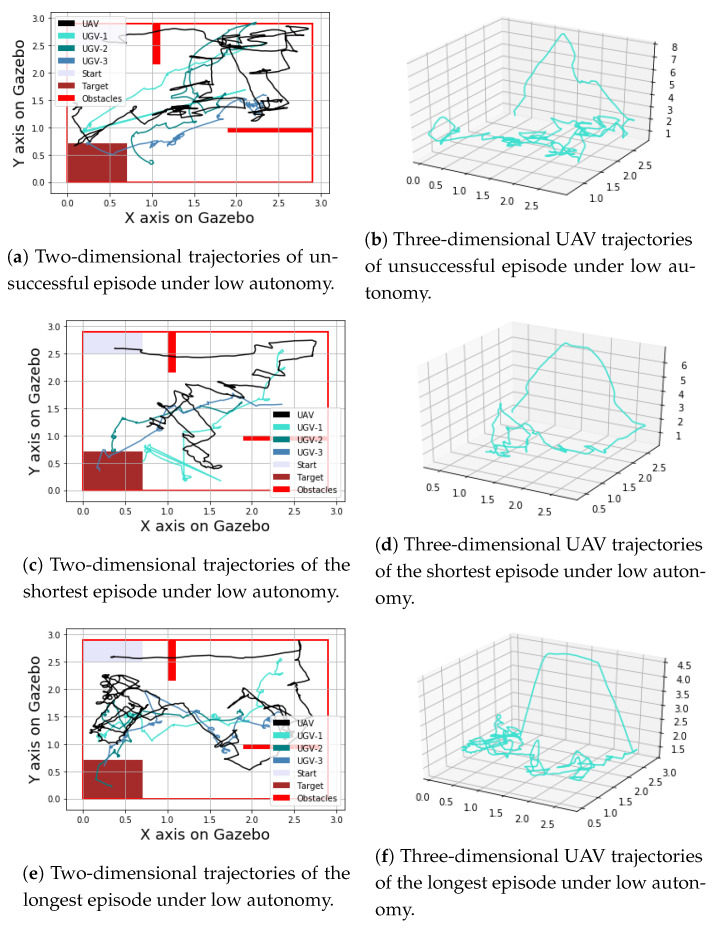
Examples of trajectories from episodes with low level of autonomy.

**Figure 12:**
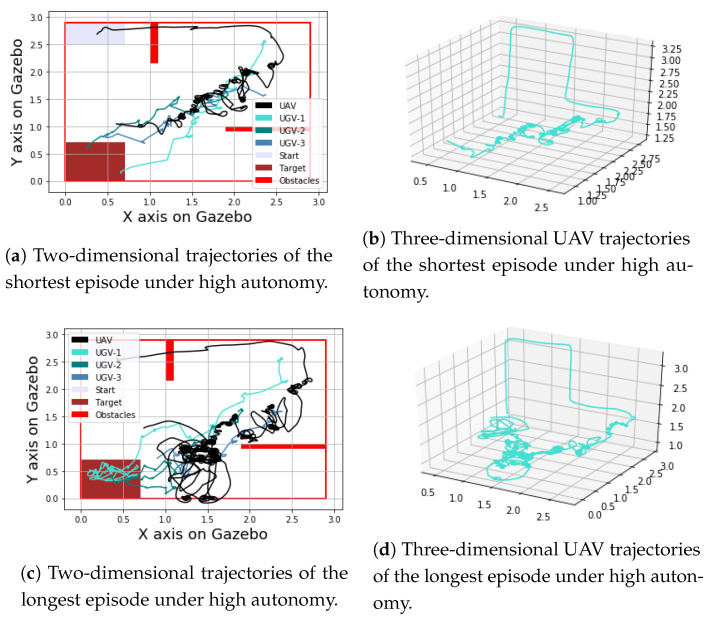
Examples of trajectories from episodes with high level of autonomy.

Looking at energy efficiency, the distance travelled by the UGVs is slightly shorter for the low level of autonomy case (mean = 16.5, std = 2.7) than the high level of autonomy case (mean = 19.3, std = 6.3); however, the difference is not statistically significant (*p* = 0.21). It is worth noting that this metric is calculated only over successful episodes. For some episodes, the low level of autonomy recorded high distances by the UGVs; however, it could not reach success, hence these episodes are excluded when calculating this metric. From these results, it is clear that the high level of autonomy results in higher success, higher time efficiency, and less workload on the human (indicated by the number of task switches). Figure 11 and Figure 12 show different examples of UGV and UAV trajectories from episodes with low and high levels of autonomy, respectively.

## 5. Discussion and Conclusions

A pressing need for swarm robotics is the design of scalable control mechanisms that can be easily deployed in realistic scenarios while imposing acceptable levels of workload on humans. Shepherding is a bio-inspired guidance technique that offers a solution to the control of large swarms using only one or few superior control agents (or dogs). A number of challenges need to be addressed to enable the adoption of shepherding in various swarm applications. First, the use of few control agents to guide the whole swarm has been shown to raise security threats as several techniques exist for the identification of swarm control agents. Once identified, these control agents can be compromised and used to spoof the swarm. Second, UAVs have been used by previous studies to perform the role of the dog due to their superior speed and navigation abilities as compared to UGVs. Nonetheless, the deployment of UAVs can be challenging in many scenarios due to the need for specialised licensing, safety pilots, and compliance with aviation regulations.

Our proposed metaverse enables us to use shepherding for controlling physical swarms while addressing these challenges. We use a symbiotic simulation environment that contains digital twins for individuals in a swarm in addition to virtual control agents. The use of virtual control agents negates the possibility of attacking the swarm by identifying them and compromising their operation. Virtual control agents also enable a simple way of guiding the swarm without requiring the use of actual physical UAVs. An additional benefit of using symbiotic simulation environments is that constraints on swarm members’ movements can be enforced by adding obstacles to the simulated environment. In the case study presented in Section 4, we showed that no trespassing areas can be easily implemented by placing walls in the simulated environment.

To manage the human workload during the interaction with the control agent, it is important to design an easy-to-use interface for authenticating human commands and to select an appropriate level of autonomy for the control agent. To enable natural communication between the human and the UAV, our case study used gestural communication due to its intuitiveness and ease of learning. Our case study investigated two levels of autonomy: the lower one required the human to fully control the UAV operation; whereas the higher one enabled the human to interact through high-level commands. Our results align with previously published results on this matter by showing that higher levels of autonomy improve performance and reduce the workload imposed on humans.

## 6. Future Work

Incorporating the human element into swarm operations is deemed necessary to account for the lack of human-like intelligence. A promising extension to our work is to utilise advanced artificial intelligence (AI) techniques to assist humans with swarm control. For example, reinforcement learning has been previously used to autonomously guide simulated swarms [14]. However, there is a lack of research on investigating the use of AI techniques in supporting human control over swarms in physical environments. Integrating such techniques within our metaverse can facilitate swarm control, particularly when considering swarm members with heterogeneous capabilities similar to those in [63]. Human interaction would still be needed for ensuring safe and ethical swarm operations and to provide necessary corrections, thus enabling learning in the AI component.

Another important research direction is evaluating the proposed metaverse with multiple UAVs. This setting will increase the number of joint behaviours performed by the UAVs and might require extending the set of commands accordingly to ensure efficient control. Along similar lines, extending the metaverse to allow simultaneous control by multiple humans/teams could prove useful for dealing with high-workload scenarios. This might require extending the interface to accommodate human-to-human coordination by incorporating multiple communication modalities (e.g., speech and gestures). 

## Figures and Tables

**Table 1:** Summary of the existing swarm control mechanisms.

Mechanism	Examples	Advantages	Limitations
Tele-operating swarm members	[16,17,18]	- Simple robot controller	- High human to robot ratio - Exponential increase in workload
Behaviour selection	[19,20,21,22,23]	- Easily used by novice users	- All behaviours must be programmed ahead - Cannot respond to unexpected events - Sensitive to timing of switching
Parameter selection	[24,25,26]	- Flexible swarm behaviours	- Not suitable for online use
Environmental signals	[19,26,27,28]	- Enables spatial control	- Difficult to use due to indirect control - Low performance for large swarms
Controlling few swarm members	[29,30,31,32,33,34,35,36]	- Flexible human interventions - Most validated HSI mechanism	- Adversarial attack by compromising swarm leader - Limited ability for controlling different swarm grouping configurations

**Table 2:** Experimental results.

	SR	Steps	Switches	Distance Travelled by UGVs
	(%)	μ±σ	μ±σ	μ±σ
Low autonomy	75	6638 ± 1132	221 ± 66	16.5 ± 2.7
High autonomy	100	5121 ± 1381	40 ± 13	19.3 ± 6.3

## Data Availability

The data presented in this study are available on request from the corresponding author.
